# Exploration of vitamin D metabolic activity-related biological effects and corresponding therapeutic targets in prostate cancer

**DOI:** 10.1186/s12986-024-00791-2

**Published:** 2024-04-02

**Authors:** Lei Ding, Yong Wang, Zhentao Tang, Chenbo Ni, Qian Zhang, Qidi Zhai, Chao Liang, Jie Li

**Affiliations:** 1 Department of Urology, The First Affiliated Hospital of Nanjing Medical University, 300 Guangzhou Road, 210009 Nanjing,, China; 2 Department of Urology, The Affiliated Wuxi People’s Hospital of Nanjing Medical University, 299 Qingyang Road, 214023 Suqian, China

**Keywords:** Vitamin D, Prostate cancer, Prognostic model, Apolipoprotein E

## Abstract

**Background:**

Previous studies have unequivocally demonstrated that the vitamin D (VD) metabolism pathway significantly influences prognosis and sensitivity to hormone therapy in prostate cancer (PCa). However, the precise underlying mechanism remains unclear.

**Methods:**

We performed molecular profiling of 1045 PCa patients, leveraging genes linked to VD synthesis and VD receptors. We then identified highly variable gene modules with substantial associations with patient stratification. Subsequently, we intersected these modules with differentially expressed genes between PCa and adjacent paracancerous tissues. Following a meticulous process involving single-factor regression and LASSO regression to eliminate extraneous variables and construct a prognostic model. Within the high-risk subgroup defined by the calculated risk score, we analyzed their differences in cell infiltration, immune status, mutation landscape, and drug sensitivity. Finally, we selected Apolipoprotein E (APOE), which featured prominently in this model for further experimental exploration to evaluate its potential as a therapeutic target.

**Results:**

The prognostic model established in this study had commendable predictive efficacy. We observed diminished infiltration of various T-cell subtypes and reduced expression of co-stimulatory signals from antigen-presenting cells. Mutation analysis revealed that the high-risk cohort harbored a higher frequency of mutations in the TP53 and FOXA genes. Notably, drug sensitivity analysis suggested the heightened responsiveness of high-risk patients to molecular inhibitors targeting the Bcl-2 and MAPK pathways. Finally, our investigation also confirmed that APOE upregulates the proliferative and invasive capacity of PCa cells and concurrently enhances resistance to androgen receptor antagonist therapy.

**Conclusion:**

This comprehensive study elucidated the potential mechanisms through which this metabolic pathway orchestrates the biological behavior of PCa and findings hold promise in advancing the development of combination therapies in PCa.

**Supplementary Information:**

The online version contains supplementary material available at 10.1186/s12986-024-00791-2.

## Introduction

As the most prevalent malignant tumor affecting men worldwide, prostate cancer (PCa) has garnered significant attention from urological researchers, focusing on its pathogenesis, mechanisms of drug resistance, and treatment modalities [1]. Among these, hormone therapy stands out as the primary therapeutic approach for PCa patients who are no longer eligible for surgery or of advanced age, demonstrating substantial efficacy, particularly in cases of primary PCa [2]. Regrettably, a common occurrence following multiple cycles of hormone therapy is the transition of hormone-sensitive PCa to castration-resistant PCa, characterized by low serum testosterone levels concurrent with activation of the androgen receptor (AR) signaling pathway [3]. Although the 5-year overall survival rate exceeds 95% for both localized and locally advanced PCa, this rate significantly drops to 32.3% in PCa patients with an extensive burden of bone or visceral metastases [4]. The imperative challenge remains in the delay or reversal of castration resistance in PCa. Furthermore, even among PCa patients meeting the criteria for radical treatment, the biochemical recurrence rate post-treatment can reach 27-53%, closely intertwined with the utilization of hormone therapy and subsequent prognosis [5]. Thus, the quest for prognostic signatures that predict disease-free survival (DFS) in PCa is of paramount significance.

The synthesis and functional orchestration of vitamin D (VD) involve the conversion of 7-dehydrocholesterol into cholecalciferol (VD3), hydroxylation processes within the liver and kidney, VD hormone transportation, and binding with the vitamin D receptor (VDR). These intricate processes closely involve cytochrome P450 enzymes, VD-binding proteins, retinol X receptors, and various other molecules [6]. Genes associated with these molecular components, which intricately regulate them, are widely expressed in human tissues, suggesting their potential impact on diverse biological processes in malignancies originating from the corresponding tissues. In recent years, the anticancer properties of the VD metabolic pathway in PCa have progressively come to light. Elements such as VD itself and its associated enzymes, including VDR, have been shown to influence PCa proliferation, invasion, and resistance to treatment [7–10]. Retrospective clinical studies have underscored the significant correlation between abnormal serum vitamin levels and PCa prognosis [11, 12]. Notably, a single-cell sequencing investigation conducted in genetically engineered Pten^(i)pe−/−^ mice revealed that VD analogs can induce apoptosis in senescent prostatic intraepithelial neoplasia and diminish the infiltration of myeloid-derived suppressor cells within the prostate [13]. Nevertheless, the underlying mechanisms responsible for these antitumor phenomena remain unclear. Hence, it is imperative to delve into genes related to the VD metabolic pathway to prognosticate DFS in PCa and formulate precision medicine strategies.

This article presents a comprehensive genomic analysis, leveraging genes related to the VD metabolic activity, along with transcriptome data and clinical characteristics from a cohort of 1045 PCa patients extracted from The Cancer Genome Atlas (TCGA), cBioPortal, and Gene Expression Omnibus (GEO) databases. Drawing upon these findings, we embarked on an initial exploration of the potential mechanisms through which the VD metabolic activity influences PCa, thereby laying a foundation for the development of therapeutic targets related to VD metabolism.

## Methods and materials

### Extraction and preprocessing of transcriptome and clinical data of patients

In addition to the TCGA database, we conducted a meticulous screening process involving the cBioPortal and GEO databases, guided by the following rigorous criteria: (1) A prerequisite was the presence of a platform annotation file with an ample number of probes, exceeding 45,000, along with the assumption that all probe values within each sample exceeded zero. (2) The essential clinical characteristics and prognostic information of the patients must be accessible. (3) Moreover, datasets with a sample count of no less than 40 were deemed suitable for inclusion in our analysis.

Ultimately, the following datasets met our stringent criteria: TCGA-Prostate Adenocarcinoma (PRAD) from TCGA database, Prostate Adenocarcinoma datasets from the German Cancer Research Center (DKFZ-PRAD), Memorial Sloan Kettering Cancer Center (MSKCC-PRAD), and Stand Up to Cancer Prostate Cancer Foundation (SU2C_PCF-PRAD) from the cBioPortal database, as well as GSE116918, GSE46602, GSE70768, and GSE70769 from the GEO database. Subsequent extraction of transcriptome and clinical data was meticulously executed using the Perl programming language.

Before commencing further analyses, several preparatory steps were performed. Samples with incomplete clinical data were excluded from the dataset. Additionally, genes exhibiting low expression levels across the majority of the samples were pruned. To facilitate comparative analysis with microarray data, Fragments Per Kilobase of exon model per million mapped fragments (FPKM values) from transcriptome sequencing data were meticulously converted into Transcripts Per Kilobase of exon model per million mapped reads (TPM values) [14]. Subsequently, the combat function within the R software package sva was deployed to alleviate the batch effects inherent in the datasets [15]. In the culmination of these rigorous procedures, we successfully derived an mRNA expression matrix along with pertinent clinical information encompassing 1045 PCa patients.

### Acquisition of VD metabolic pathway gene set and differential genes

Gene sets pertinent to the VD metabolic pathway are readily accessible within the Molecular Signatures Database (MSigDB) (V7.5.1) [16]. Additionally, for the purpose of differential analysis (|logFC| > 1, *P* < 0.05) aimed at identifying differentially expressed genes between tumor and normal tissues, we harnessed the capabilities of the R software package Limma (version 3.40.6) [17].

### Weighted gene co-expression network analysis (WGCNA) and Enrichment analysis

We stratified the patients into distinct groups based on molecular typing to discern the varying levels of VD metabolic activity. Subsequently, we employed the R software package WGCNA to probe gene sets closely associated with VD activity [18]. Using this method, gene expressions sharing analogous patterns were clustered into co-expression modules, allowing us to identify modules that were significantly correlated with the VD metabolic activity phenotype (|r|≥0.3, *P* < 0.05).

Upon ascertaining the gene modules through WGCNA analysis, we embarked on the Gene Ontology (GO) and Kyoto Encyclopedia of Genes and Genomes (KEGG) analyses to elucidate the relevant signaling pathways. Furthermore, we used the Gene Set Enrichment Analysis (GSEA) software (version 3.0) from the GSEA website, alongside the c2.cp.biocarta.v7.4. symbols.gmt subset from MsigDB for a comprehensive evaluation of pathways and molecular mechanisms associated with VD metabolic activity [16, 19]. Notably, statistical significance was established at a threshold of *P* < 0.05 and FDR < 0.25.

### Establishment, evaluation and validation of prognostic model

To construct a prognostic model involving VD metabolism-related genes, we conducted lasso regression analysis using the R software package Glmnet. The risk score was computed using the following formula: Risk score = ${\sum }_{i=0}^{n}\text{C}\text{o}\text{e}\text{f} \left(\text{i}\right) \times \text{x} \left(\text{i}\right)$. Coef (i) and x (i) represent the estimated regression coefficient and the expression of genes, respectively [20]. Subsequently, we employed both univariate and multivariate regression analyses to examine the association between prognosis and gene expression as well as the risk score. To visualize the differences in prognosis across the various groups, we employed Kaplan-Meier (K-M) survival curves. The effectiveness of the prognostic model was assessed using receiver operating characteristic (ROC) curves. Furthermore, we developed a nomogram based on multifactor regression analyses, incorporating clinical features and risk scores to depict the relative impact of these factors on prognosis [21]. To validate the efficacy of the prognostic model, we randomly selected 400 patients using the sample function in the R software for internal validation.

### Mapping of the protein–protein interaction network

The protein-protein interaction (PPI) network of proteins encoded by DEGs was analysed using the STRING database (https://string-db.org/). We then used the MCODE plug-in unit of the Cytoscape software (version: 3.9.1) to extract the PPI subnet. Cytohubba plug-in of cytoscape was used to calculate the centrality of genes using the maximal clique centrality (MCC). Through this method, the MCC values of each gene in the network were obtained, and genes with MCC values greater than 10 were defined as hub genes and highlighted in red in the PPI network [22]. 25,521,941 The PPI network was also visualised using the Cytoscape software.

### Evaluation of tumor microenvironment (TME) infiltration and immune status

We employed the ESTIMATE algorithm within the R software package Estimate to predict various aspects of the tumor microenvironment, including tumor purity, the overall infiltration of cells (estimate score), stromal cells (stromal score), and immune cells (immune score) [23]. Additionally, we utilized the CIBERSORT algorithm available in the R software package IOBR to estimate the infiltration of diverse immune cell subtypes and assess immune function-related scores for the sampled data [24]. To elucidate the relationships between different types of immune cells in distinct groups, we conducted Pearson’s correlation coefficient analyses to quantify the degree of correlation among these variables.

### Calculation of tumor mutation burden (TMB) and microsatellite instability (MSI)

Using the Perl programming language, we extracted mutation data from samples within the TCGA dataset. The data were subsequently used to compute the TMB score for each sample, denoting the number of mutations per million bases. Furthermore, we employed the R software package PreMSIm to calculate the MSI score for samples within TCGA dataset [25].

### Drug sensitivity evaluation

We accessed the CellMiner online database at https://discover.nci.nih.gov/cellminer/home.do to examine the correlation between the efficacy of pharmacological treatments and gene expression levels [26]. From this database, we obtained DTP NCI-60 data that included average z-scores along with the corresponding RNA-seq datasets. Subsequently, we compared the predicted half-maximal inhibitory concentrations (IC50) of various drugs between high-risk and low-risk samples using a group t-test.

### Statistical analysis

We normalized the TPM values within the matrix by applying a log_2_(X + 1) transformation using the R software package Limma. The criteria for statistical significance in all t-tests and correlation analyses were set at *P* < 0.05. Statistical analyses and figures were created using R software and GraphPad Prism 8.

## Results

### Screening of VD metabolic activity related genes in PCa

**Fig. 1:**
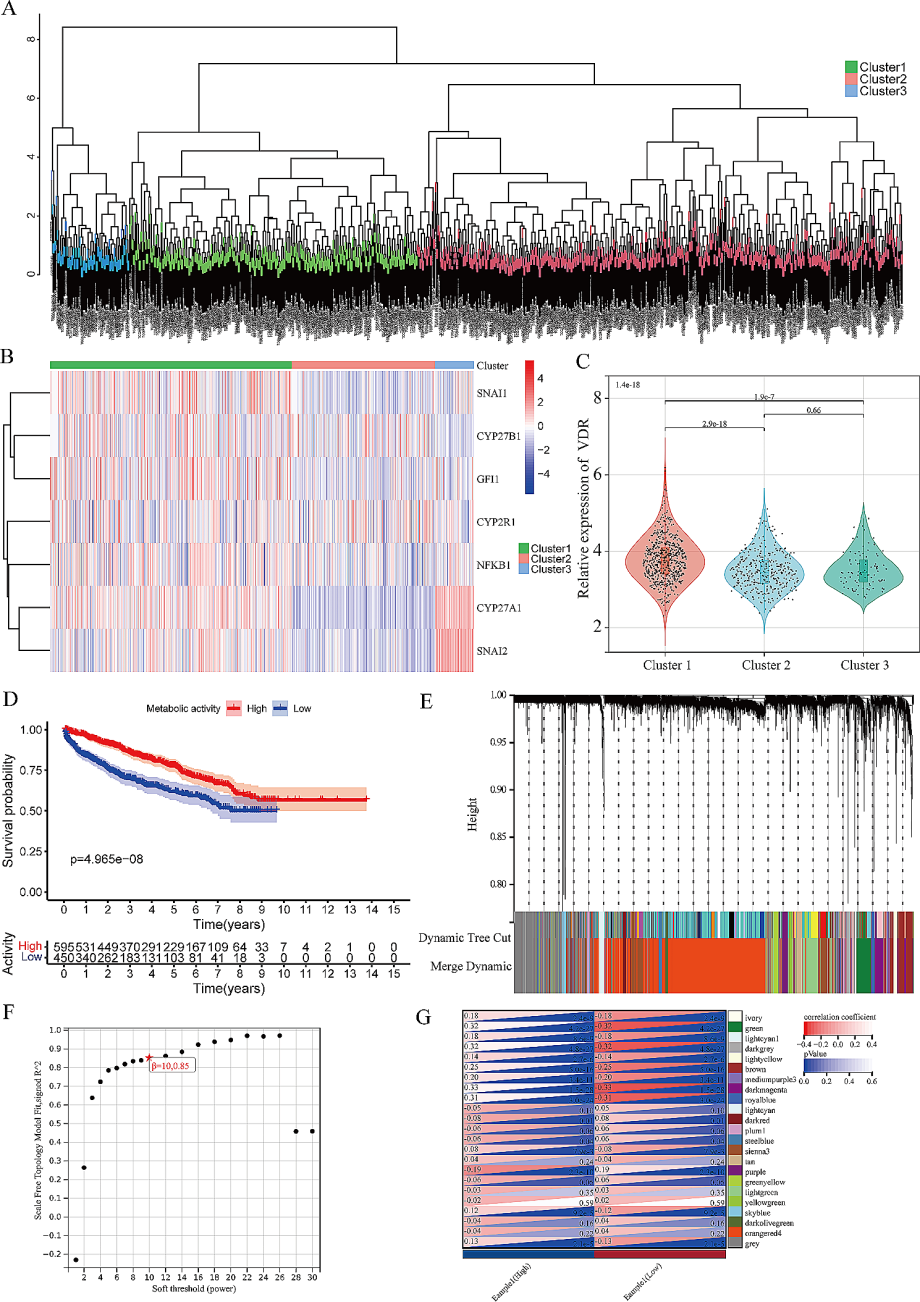
Exploration of gene modules related to vitamin D metabolic activity. **A**: The cluster tree of 1045 patients with prostate cancer enrolled in the study, colored based on clustering. **B**: The expression heatmap of vitamin D synthesis related genes in all tissue samples, colored based on relative expression levels. **C**: Violin plot of the relative expression of vitamin D receptors in all tissue samples. The expression levels of different groups were compared by group T test. **D**: The Kaplan-Meier curves of disease-free survival of patients in different vitamin D activity groups, and log-rank test was to compare the difference in prognosis between the two groups. **E**: Results of gene module merging based on clustering and dynamic pruning methods. Each vertical line indicates a gene and each branch represents an expression module of highly interconnected genes. Below the dendrogram, different modules are given different colors. Gray indicates that genes are outside all modules. **F**: Analysis of network topology for the soft-threshold power. **G**: The correlations of gene modules with multiple clinical features, colored by *P* values and correlation coefficients

In this study, 1045 patients from eight datasets sourced from TCGA, cBioPortal, and GEO databases were included. The clinical characteristics of the patients are detailed in Table 1 (Method). After rigorous batch effect removal, heterogeneity between the datasets was substantially mitigated (Figure.S1A, B). Initially, we used the expression similarity of VD metabolic pathway genes from the MsigDB database to classify these patients into three distinct clusters (Fig. 1A). Notably, patients in cluster 1 exhibited higher overall expression levels of VD metabolism pathway genes, and the expression of VDR was notably elevated compared to the other clusters (Fig. 1B, C). Consequently, we inferred that patients within cluster 1 manifested relatively heightened VD metabolic activity within their PCa cells. Building on this distinction, we categorized the patients into two groups: those with high VD metabolic activity (cluster 1) and those with low activity (clusters 2 and 3). This classification was further supported by Kaplan-Meier survival analysis, which conclusively indicated that patients with high VD metabolic activity exhibited significantly improved DFS compared to those with low activity, consistent with previous research findings that underscored the negative correlation between VD metabolism and PCa progression (Fig. 1D).

**Table 1:** The clinical characteristics, pathological characteristics and follow-up outcomes of the patients included in the training set

	Group	Number (n, %)	Total
Database	TCGA	475 (45.5%)	
	cBioPortal	222 (21.2%)	
	GEO	348 (33.3%)	1045
Age	≤ 65	508 (48.6%)	
	>65	352 (33.7%)	860
Gleason score	4–6	157 (15.0%)	
	7–8	669 (64.0%)	
	9–10	217 (20.8%)	1043
PSA value	4<PSA ≤ 10	148 (14.2%)	
(ng/ml)	10<PSA ≤ 20	145 (13.9%)	
	PSA>20	151 (14.4%)	444
T stage	T1	140 (13.4%)	
	T2	404 (38.7%)	
	T3	451 (43.2%)	
	T4	17 (1.6%)	1012
M stage	M0	458 (43.8%)	
	M1	2 (0.2%)	460
N stage	N0	347 (33.2%)	
	N1	74 (7.1%)	421
Survival status	Recurrence	282 (27.0%)	
(DFS)	No recurrence	763 (73.0%)	1045

TCGA: The Cancer Genome Atlas; GEO: Gene Expression Omnibus; PSA: prostate-specific antigen; DFS: disease free survival

Additionally, we conducted WGCNA analysis to elucidate the gene sets associated with this molecular classification (Fig. 1E). In this analytical endeavor, we set the soft-thresholding power to 10, cut height to 0.85, and minimal module size to 40 (Fig. 1F). The results of this analysis yielded four gene modules that exhibited robust correlations with VD metabolic activity for subsequent investigation (|r| ≥ 0.3, *P* < 0.05) (Fig. 1G, Figure.S2).

### Exploration of signaling pathways related to VD metabolic activity

**Fig. 2:**
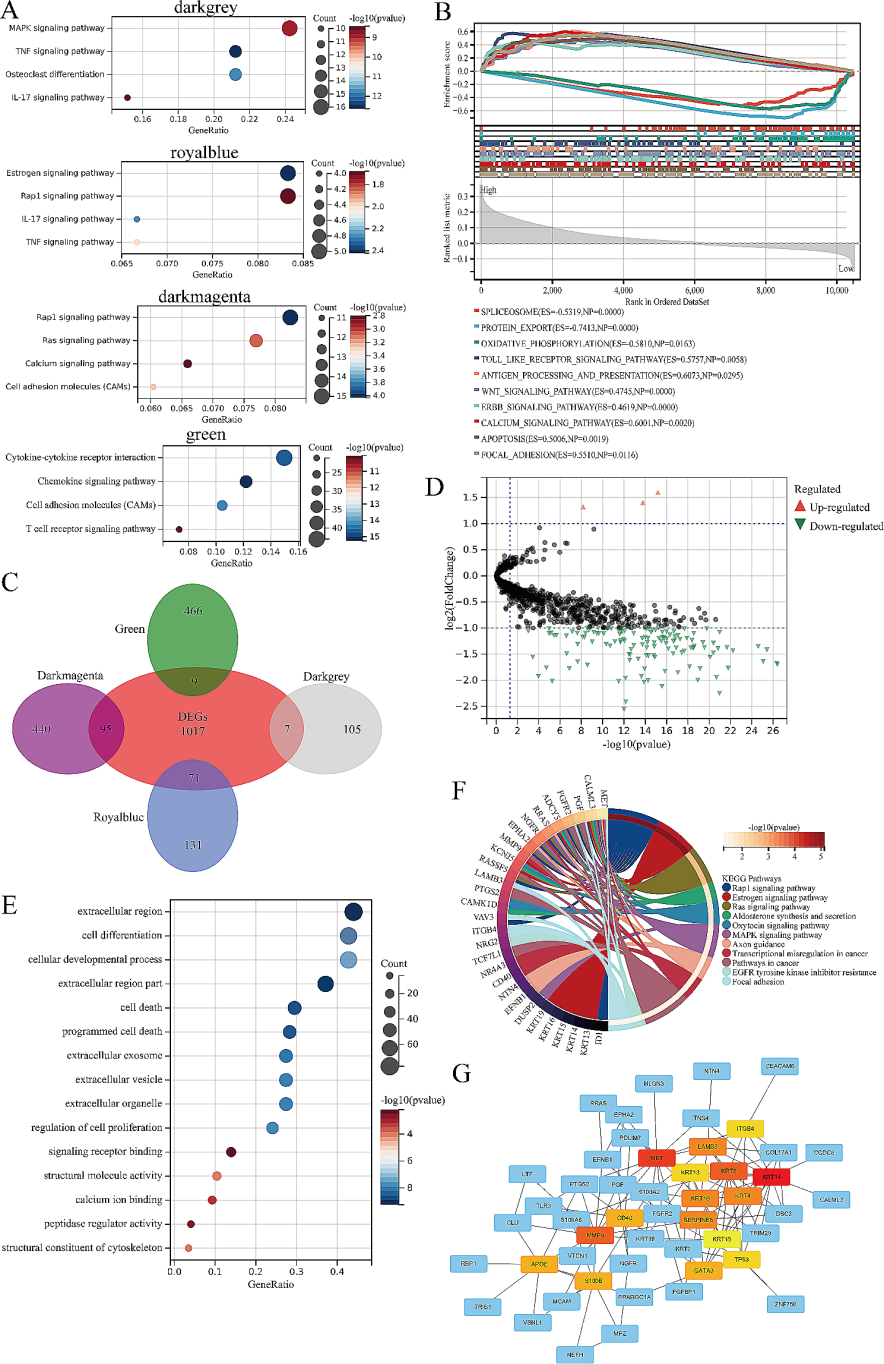
Signaling pathways related to by vitamin D metabolic activity. **A**: The bubble diagram of the results of KEGG analysis of gene modules related to vitamin D metabolic activity. **B**: Presentation of the results of GSEA analysis based on vitamin D metabolic activity groups. **C**: Venn diagram of intersection of vitamin D metabolism-related gene modules DEGs between prostate cancer and paracancer tissue. **D**: Volcanic map of intersection genes in Fig. 2 C, and the differential genes (|Fold Chage| ≥ 2, *P* < 0.05) were marked in color. **E**: The bubble diagram of the results of GO analysis of intersection genes in Fig. 2C. **F**: Cyclic graph of the results of KEGG analysis of intersection genes in Fig. 2 C, colored by signaling pathways. **G**: Protein interaction network diagram of intersection genes in Fig. 2 C. The red ones are the hub genes, and the redder the color, the more proteins interact with them. DEGs: differentially expressed genes

To gain insight into the biological processes associated with the previously identified genes that were highly correlated with VD metabolic activity, we conducted KEGG enrichment analysis (Fig. 2A). This analysis revealed significant enrichment of signaling pathways relevant to tumorigenesis, including the MAPK, TNF, Ras, and Rap1 pathways. Notably, the estrogen signaling pathway, known to counteract androgen signaling, was also prominently enriched within the royal blue gene module. Subsequently, we conducted GSEA based on the earlier grouping of VD activity to delve further into the signaling pathways associated with this phenotype (Fig. 2B). Our findings indicated that in the high VD activity group, there was increased expression of tumor-related signals, such as WNT and ERBB, alongside immune process-related signals encompassing antigen processing, presentation, and toll-like receptors. Conversely, in the low-activity group, the signal linked to oxidative phosphorylation was elevated.

Next, we intersected the genes within these modules with the differentially expressed genes between PCa and adjacent tissues to identify potential genes implicated in regulating PCa onset and progression (Fig. 2 C). Interestingly, the majority of these intersecting genes exhibited lower expression in PCa (Fig. 2D). In the results of KEGG enrichment analysis for these intersecting genes, the Rap1, Ras, and MAPK signaling pathways were significantly enriched. Furthermore, signals associated with tumor metastasis, such as EGFR tyrosine inhibitor resistance and focal adhesion, were strongly correlated with these genes (Fig. 2E). GO analysis revealed that these genes primarily operate within the extracellular matrix and play roles in essential biological processes such as cell differentiation, proliferation, and apoptosis. They were also intricately connected to intercellular communication signaling pathways, including exosomes (Fig. 2F). Furthermore, protein interaction network analysis of these intersection genes revealed that markers of basal cells (KRT5, KRT14, TP63) and the stem cell-related gene CD40 served as hub genes within this interaction network (Fig. 2G).

### Establishment, evaluation and validation of the prognostic model related to VD metabolic activity

**Fig. 3:**
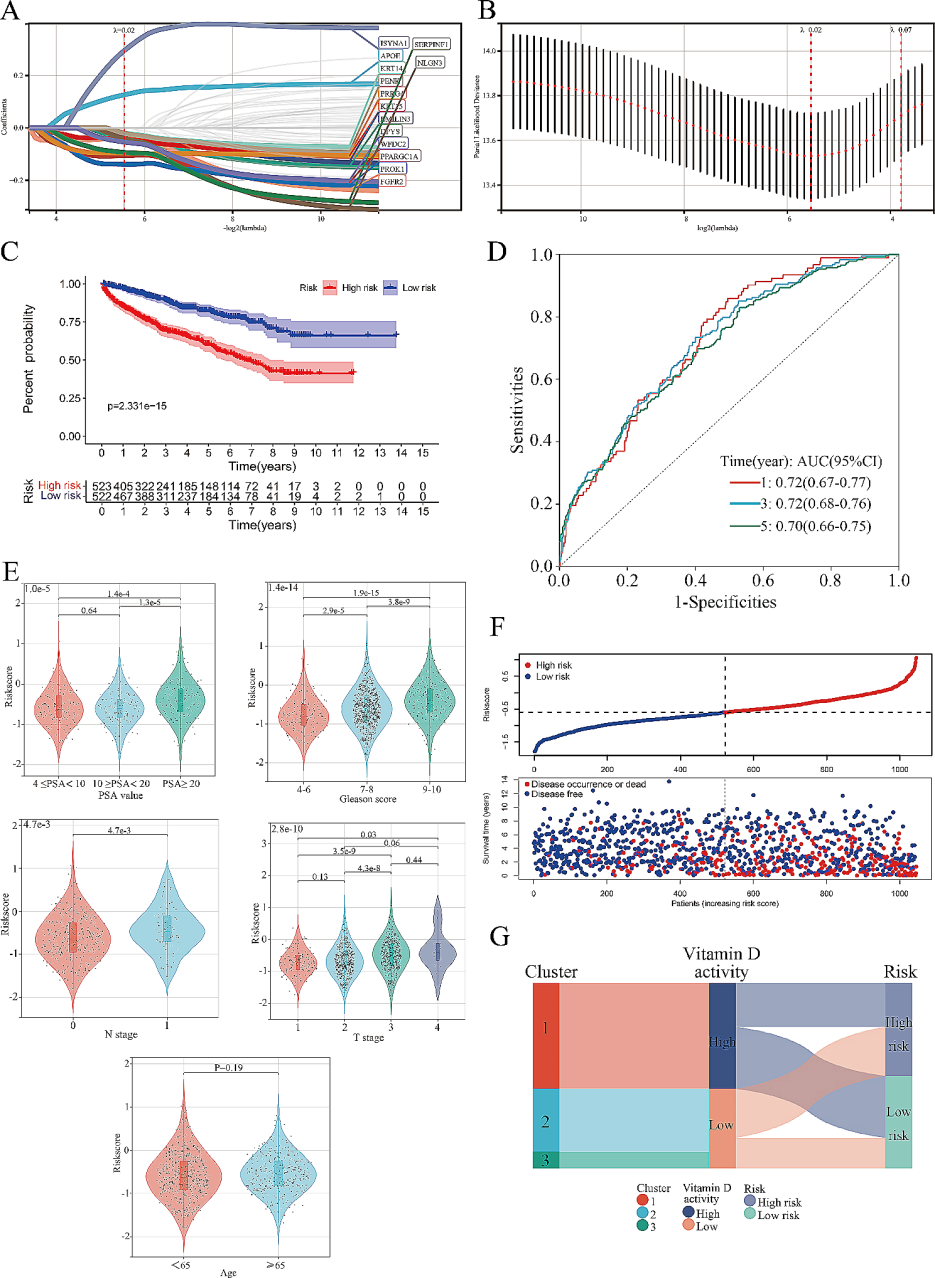
Construction of the prognostic model related to vitamin D metabolic activity. **A**: LASSO regression analysis of genes related to vitamin D metabolic activity. **B**: Optimal penalty parameter λ (0.02) identified by tenfold cross-validation. **C**: The Kaplan-Meier curves of disease-free survival of patients in the high- and low-risk groups, and log-rank test was to compare the difference in prognosis between the two groups. **D**: ROC curves of 1,3,5 years for the prognostic model related to vitamin D metabolic activity. **E**: Violin diagrams of the risk scores in different subgroups. The risk scores of different groups were compared by group T test. **F**: The scatter plot of the patient’s survival time (top) and the distribution of risk scores estimated by the prognostic model (bottom). **G**: The Sankey diagram exhibits the distribution of enrolled patients in clustering, vitamin D metabolic activity, and risk grouping. The nodes represented different classifications, and the width of nodes and traffic represented the number of patients. PSA: prostate-specific antigen

We identified genes from the gene set in Fig. 2 C that were significantly associated with DFS of PCa patients through batch univariate regression analyses (*P* < 0.05) (Figure.S3). Subsequently, we integrated the expression data of these genes with patient survival data and constructed a prognostic model linked to VD metabolic activity using Lasso-Cox regression analysis, with a lambda value of 0.02151 (Fig. 3A, B). After the elimination of genes by Lasso-Cox regression analysis, 14 genes were ultimately used to construct the prognosis model and the formula is as follows: Risk Score = 0.12615 * APOE − 0.04210 * DPYS − 0.03177 * EMILIN3–0.03915 * FGFR2 + 0.29453 * ISYNA1–0.02007 * KRT14–0.04128 * KRT15–0.04074 * NLGN3–0.02005 * PENK − 0.10676 * PPARGC1A − 0.13883 * PROK1–0.09616 * PRRG4–0.09378 * SERPINF1–0.05412 * WFDC2. Subsequently, we divided the patients into high- and low-risk groups based on the median risk score and generated Kaplan-Meier curves (Fig. 3 C). The results indicated that patients in the low-risk group exhibited significantly improved DFS compared with those in the high-risk group. Further evaluation of the prognostic model performance revealed that the area under the ROC curve for 1, 3, and 5 years exceeded 0.70, underscoring its robust predictive efficacy (Fig. 3D). Furthermore, we explored the relationship between this risk score and various clinical characteristics, revealing a significant increase in the risk score with higher PSA values, Gleason scores, and tumor stages (Fig. 3E). The scatter plot depicting the risk scores and endpoint events highlighted a noticeable increase in the incidence of endpoint events in the high-risk group (Fig. 3F). The Sankey diagram, which amalgamated clustering, VD activity grouping, and risk grouping, indicated that the majority of patients in the high VD activity group were classified into the low-risk group, consistent with the findings of the survival analysis (Fig. 3G).

**Fig. 4:**
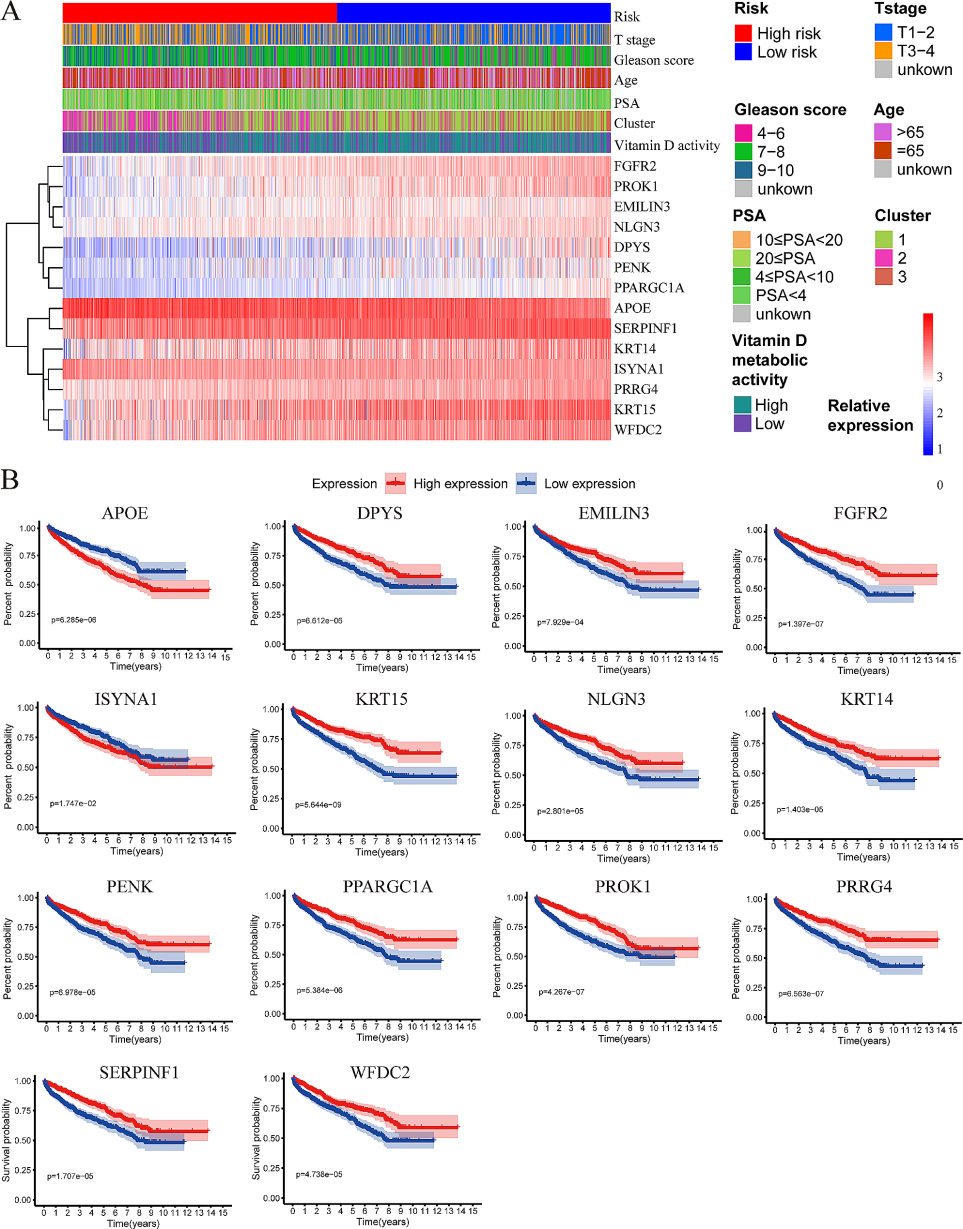
Expression distribution and prognostic value of prognostic model-related genes. **A**: The heatmap exhibit the clinical characteristics, clustering, vitamin D metabolic activity grouping and expression levels of genes involved in the prognostic model of patients in the high- and low-risk groups. **B**: The Kaplan-Meier curves of disease-free survival of patients with different expression levels of genes involved in the prognostic model. PSA: prostate-specific antigen

To provide a comprehensive visualization of the associations between risk scores, clinical features, VD activity groups, and the expression levels of genes included in the model, we presented these data in a heat map (Fig. 4A). Evidently, patients in the high-risk group generally exhibited higher T stage (X-squared = 41.356 *P* < 0.001), Gleason scores (X-squared = 49.926, *P* < 0.001), and PSA levels (X-squared = 24.142, *P* < 0.001). Moreover, a substantial proportion of high-risk patients belonged to the low VD activity group (clusters 2 and 3) (X-squared = 41.865, *P* < 0.001). We also generated Kaplan-Meier curves for the genes associated with the model (Fig. 4B). These curves revealed that only Apolipoprotein E (APOE) and ISYNA1 were classified as risk genes, while the remaining genes were protective. In conjunction with the findings discussed in the Introduction section, we postulate that dysregulation of VD metabolism may be prevalent in high-risk groups.

**Fig. 5:**
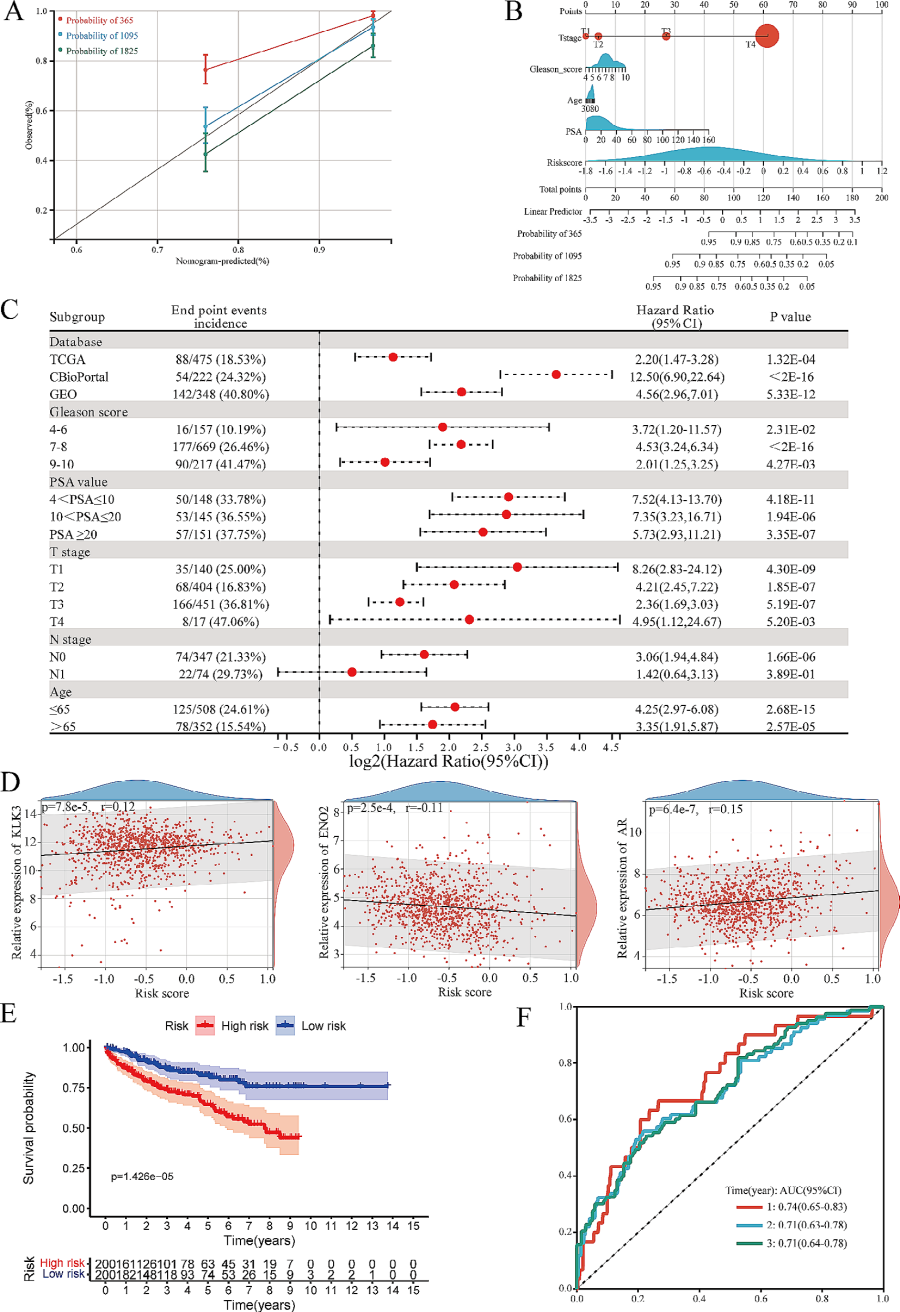
Evaluation of prognostic efficacy and stability of the risk score. **A**: The nomogram constructed based on clinicopathological parameters and the risk score and predict the 1, 3 and 5-year DFS of patients. **B**: Calibration curves exhibit the consistency between the observed and predicted 1, 3 and 5-year DFS of patients. **C**: The forest map exhibits the results of univariate regression analyses of the risk score in different subgroups. **D**: Point plots of the results of pearson correlation coefficient analysis between gene expression and the risk score. **E**: The Kaplan-Meier curve of disease-free survival of patients in the high- and low-risk groups from the validation set, and log-rank test was to compare the difference in prognosis between the two groups. **F**: ROC curves of 1,3,5 years for the prognostic model related to vitamin D metabolic activity in the validation set. DFS: disease free survival

Subsequently, we integrated multiple clinical features with risk scores to enhance the accuracy of prognosis prediction for patients with PCa. The calibration curve showed that the predicted 3, 5-year DFS were highly consistent with the actual DFS (Fig. 5A) and the corresponding nomogram underscored the substantial contribution of risk score in predicting patient outcomes (Fig. 5B). To assess the stability of this prognostic model, a univariate regression analysis of the risk scores was conducted in different subgroups. The results revealed that except for the N1 subgroup, the risk score remained a statistically significant risk factor in all subgroups (Fig. 5 C). Moreover, internal validation involving a randomly selected subset of 400 patients from the dataset reaffirmed the prognostic model’s robust predictive efficacy, as indicated by the K-M curve for DFS (Fig. 5D) and the ROC curve (Fig. 5E).

### Effects of VD metabolic activity related genes on the TME of PCa

**Fig. 6:**
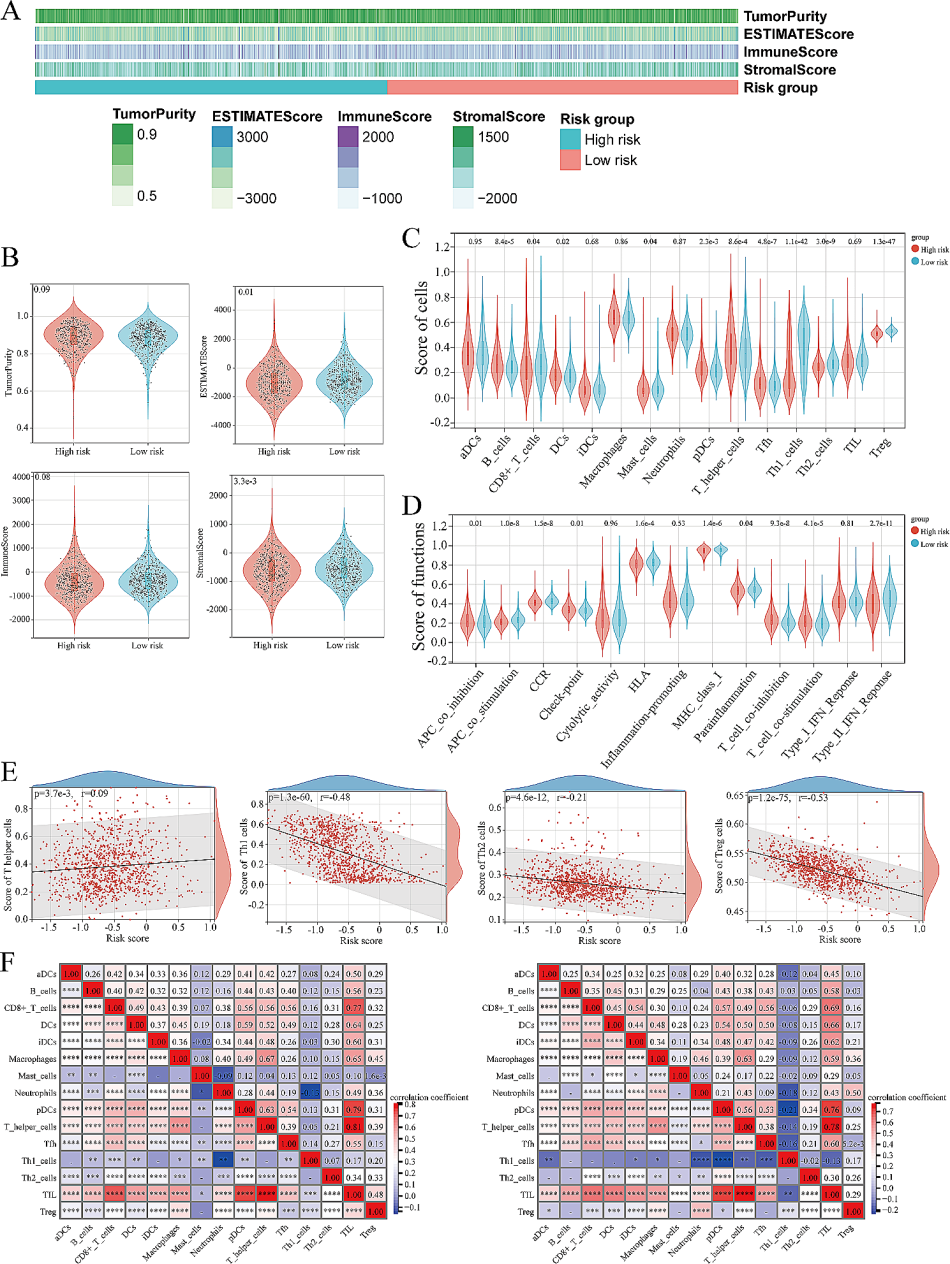
The effect of vitamin D metabolic activity related genes on TME. **A**: The heatmap exhibit the estimated TME related scores of patients in the high- and low-risk groups. **B**: Violin diagrams of TME related scores in the high- and low-risk groups, which compared by group T test. **C & D**: Violin diagrams of immune cell infiltration related scores and immune function related scores in the high- and low-risk groups, which compared by group T test. **E**: Point plots of the results of pearson correlation coefficient analysis between the risk score and immune cell infiltration related scores. **F**: Results of correlation analysis of various immune cells infiltrated in tumor tissues of the high- (left) and low-risk (right) groups. *P < 0.05, **P < 0.01, ***P < 0.001, ****P < 0.0001. aDCs: activated dendritic cells; iDCs: immature dendritic cells; pDCs: plasmacytoid dendritic cells; Tfh: follicular helper T cells; TIL: tumor-infiltrating lymphocyte; Treg: regulatory T cell; APC: antigen presenting cell; CCR: CC chemokine receptor; HLA: human leukocyte antigen; MHC: major histocompatibility complex; IFN: interferon

Previous enrichment analyses have shown that VD metabolism-related genes predominantly operate within the extracellular matrix and play widespread roles in regulating cellular proliferation, intercellular communication, and other vital biological processes. Consequently, we postulated that these genes may exert a substantial influence on the TME of PCa. We estimated the tumor purity and cell infiltration scores for each sample, visually represented in the heatmap (Fig. 6A). Subsequently, we compared the differences in cell infiltration within the TME between the high- and low-risk groups. Our findings indicated that patients in the high-risk group exhibited lower levels of cell infiltration within the tumor tissue (*P* = 0.01). This discrepancy appears to be primarily driven by the reduced infiltration of stromal cells (*P* = 3.3e-3) (Fig. 6B). Furthermore, we conducted a comparative analysis of the infiltration of various immune cells and expression levels of immune-related gene sets within the TME of PCa between the high- and low-risk groups. The results revealed significant differences in the infiltration of B cells, CD8 + T cells, dendritic cells (DCs), and other immune cells (Fig. 6 C). Additionally, immune processes related to antigen presentation exhibited increased activity in the low-risk group, whereas T-cell-related immune processes displayed heightened activity in the high-risk group (Fig. 6D). Correlation analyses further highlighted that the risk score was positively correlated with the infiltration score of T helper cells but significantly negatively correlated with the infiltration scores of Th1, Th2, and Treg cells (Fig. 6E). Moreover, we conducted correlation analyses involving various immune cell types within the high- and low-risk groups (Fig. 6F). Interestingly, in both groups, tumor-infiltrating lymphocytes exhibited significant positive correlations with various immune cells, including T cells, DC, and macrophages. Collectively, these analyses suggested that there may be significant biological differences in the TME of PCa between the high- and low-risk groups.

### Association between VD metabolic activity related genes and mutation landscape of PCa

**Fig. 7:**
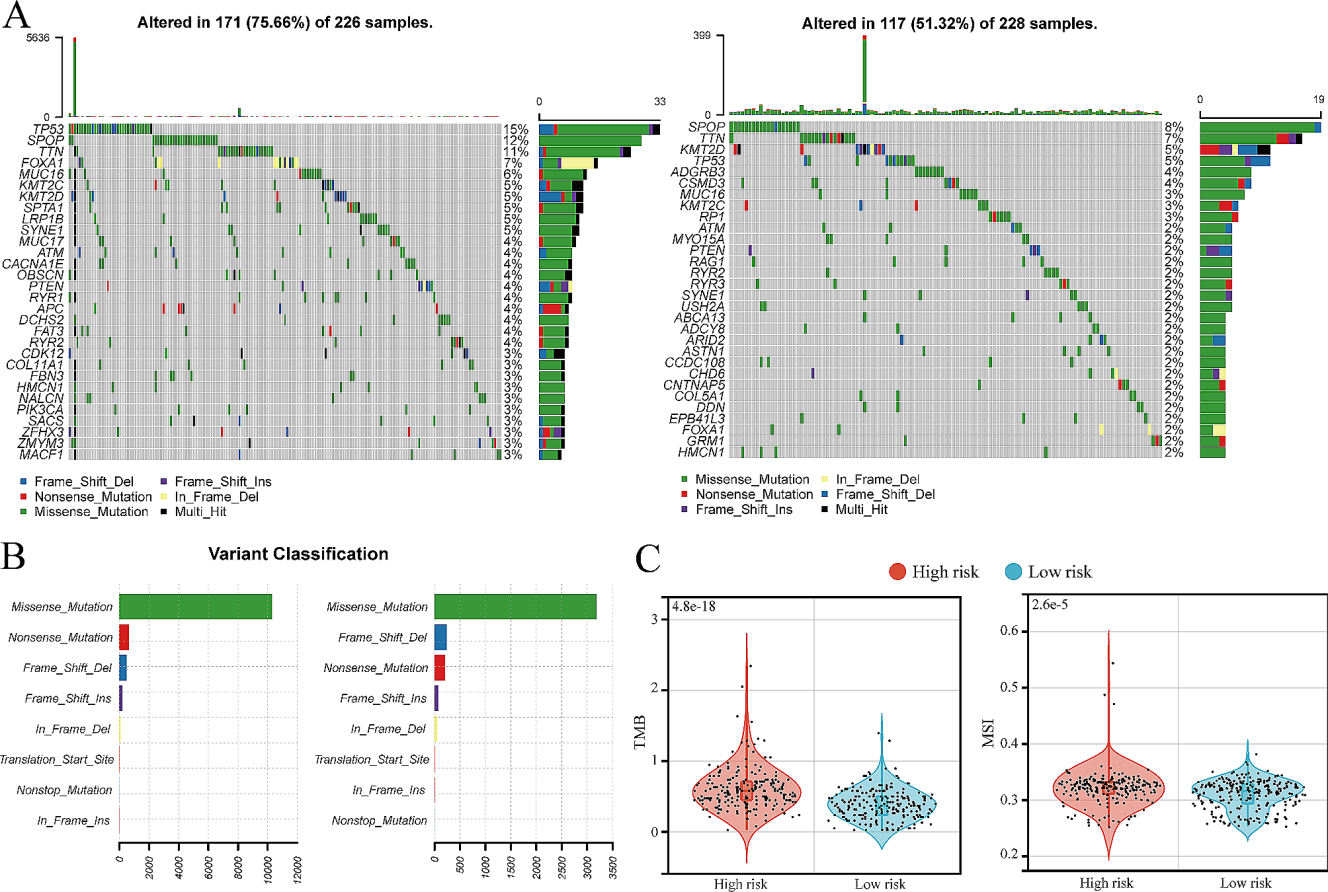
Association between genes related to vitamin D metabolic activity and tumor mutations. **A**: Mutation landscape of genes with high mutation rate in prostate cancer in the high- (left) and low-risk (right) groups. **B**: Horizontal bar chart of the numbers of each variant classification in tumor samples of the high- (left) and low-risk (right) groups. **C**: Violin diagrams of TMB and microsatellite instability in the high- and low-risk groups, which compared by group T test. Del: deletion; Ins: insertion; SNP: single nucleotide polymorphisms; TMB: tumor mutation burden; MSI: microsatellite instability

We elucidated the mutation landscape of 454 patients from both the high- and low-risk groups using waterfall plots (Fig. 7A). Notably, the high-risk group exhibited a significantly higher proportion of patients with mutations than the low-risk group (X-squared = 27.968, *P* < 0.001). Furthermore, the differences in mutation rates of TP53 (X-squared = 12.155, *P* < 0.001) and FOXA1 (X-squared = 5.086, *P* = 0.024) were particularly pronounced between the high-risk and low-risk groups. When examining the primary mutation classification for both groups, we found that missense mutations predominated (Fig. 7B) Notably, the high-risk group exhibited a higher proportion of nonsense mutations, whereas the low-risk group had a higher prevalence of frameshift deletion mutations (Fig. 7B). Additionally, we compared mutation-related scores between the two groups using a t-test. The results indicated that the Tumor Mutation Burden and MSI scores were significantly higher in the high-risk group than in the low-risk group (Fig. 7 C).

### Exploration of therapeutic drugs and targets related to VD metabolic activity

**Fig. 8:**
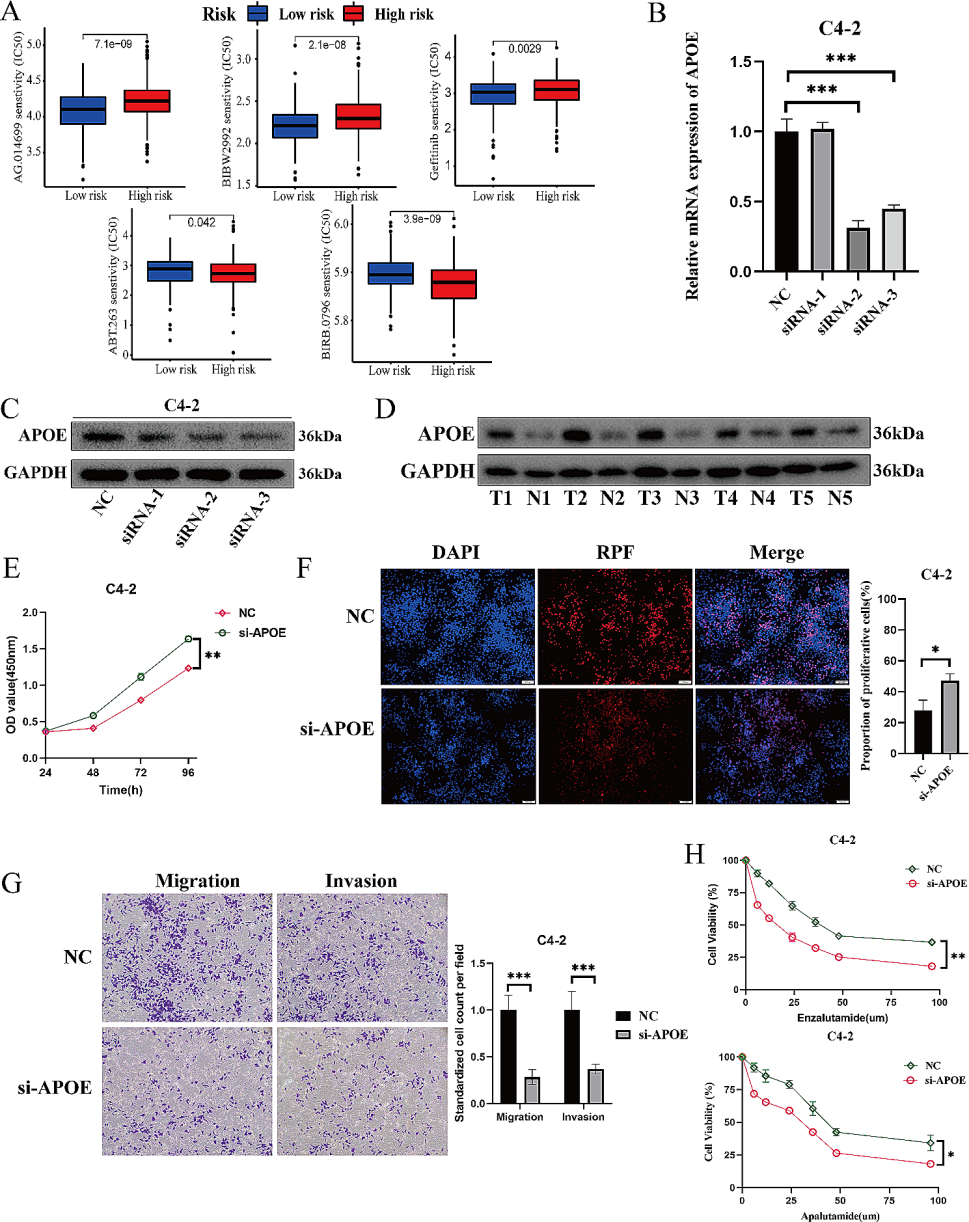
Exploration of therapeutic drugs and targets related to vitamin D metabolic activity. **A**: The predicted IC50 values of tumor cells against drugs for patients in the high- and low-risk groups, which compared by group T-test. **B & C**: The relative expression level of APOE gene in C4-2 cell lines transfected by siRNA, and the knockdown efficiency was verified by qRT-PCR (B) and western blot (C). **D**: The protein expression levels of APOE detected by western blot in five pairs of paired RCC tissues and adjacent tissues. **E**: CCK-8 proliferation assay of wild type and APOE knockdown C4-2 cell lines. **F**: EdU proliferation assay of wild type and APOE knockdown C4-2 cell lines. The images were taken at 10x. G: Transwell assay of wild type and APOE knockdown C4-2 cell lines. The images were taken at 10x. **G**: Toxicity test of enzalutamide and apalutamide on wild type and APOE knockdown C4-2 cell lines, which compared by paired T-test. OD: optical density. *P < 0.05, **P < 0.01, ***P < 0.001, ****P < 0.0001

Utilizing the CellMiner database, we conducted a preliminary screening of potential combination therapy drugs and therapeutic resistance drugs specific to the high-risk group (Fig. 8A). The predicted IC50 values for navitoclax (a Bcl-2 inhibitor, ABT.263) and doramapimod (a MAPK inhibitor, BIRB.0796) were significantly lower in the high-risk group than in the low-risk group. Conversely, for Veliparib (a PARP inhibitor, AG.014699), afatinib (an EGFR inhibitor, BIBW2992), and gefitinib (an EGFR inhibitor), the predicted IC50 values were notably higher in the high-risk group than in the low-risk group.

Furthermore, we delved deeper into potential therapeutic targets associated with VD metabolic activity. Among the genes used to construct the prognosis model, only APOE and ISYNA1 were identified as risk genes, with APOE showing a more significant prognostic difference between patients with high and low expression levels (Fig. 4B). Therefore, we consider APOE as a potential therapeutic target for PCa. This finding was corroborated by the expression levels of APOE in PCa tissues from our center, which were significantly higher than those in paracancerous tissues (Fig. 8B). We used siRNA to create an APOE-knockdown PCa cell line, C4-2, and successfully verified its effectiveness (Fig. 8 C, D). Subsequently, siRNA-2 was used for further experiments. Both the CCK-8 proliferation assay (Fig. 8E) and the EdU proliferation assay (Fig. 8F) demonstrated that APOE knockdown significantly inhibited the proliferation of PCa cells. Additionally, Transwell migration and invasion experiments indicated that APOE knockdown also had an inhibitory effect on the migration and invasion capabilities of PCa cells (Fig. 8G). Finally, we conducted cytotoxicity experiments for enzalutamide and apalutamide and found that APOE knockdown enhanced the sensitivity of PCa cells to AR antagonist therapy (Fig. 8H).

## Discussion

VD, an essential nutrient for humans, has demonstrated significance in the prevention and treatment of various cancers, including PCa [27]. Nonetheless, the intricate dysregulation mechanisms of the VD metabolic pathway and its associated signaling pathways in PCa require further exploration.

In our study, enrichment analysis revealed the involvement of Rap1, Ras, and MAPK signaling pathways in gene modules that were associatedwith VD metabolic activity and genes intersecting with the differential gene list. Rap1 and Ras signals have been shown to cooperatively initiate and sustain ERK signal transduction in various tumors, promoting tumor invasion and migration, including PCa [28, 29]. Interestingly, a study in adrenal cortical cancer highlighted that VDR can activate Rap1 signaling, resulting in antitumor proliferation effects [30]. Therefore, these signaling pathways may constitute crucial mechanisms through which the VD metabolic pathway regulates the biological behavior of tumors. Additionally, the MAPK signaling pathway, downstream of Ras signaling, has been implicated in inflammatory lung injury when VDR expression decreases [31]. Another study demonstrated that VD therapy can deactivate MAPK by upregulating MAPK phosphatase-1, thereby inhibiting the production of inflammatory cytokines in monocytes and macrophages [32]. In light of our results, we hypothesize that these signaling pathways are closely linked to the dysregulation of VD metabolism in PCa, although their underlying mechanisms warrant further investigation and verification.

Analysis of the TME revealed that the high-risk group, characterized by dysregulated VD metabolic pathways, exhibited reduced stromal cell infiltration in the TME. Previous research in cardiovascular disease has shown that active VD metabolic pathways regulate endothelial nitric oxide synthase expression or activity through transcriptome or epigenetic pathways, thereby mitigating inflammatory vascular endothelial cell damage or dysfunction [33]. In the context of Crohn’s disease, increased increases VDR protein induced by VD and inhibits fibroblast migration in damaged intestinal tissues, consequently preventing the progression of intestinal fibrosis [34]. These findings may explain the diminished stromal cell infiltration observed in the TME of the high-risk patients. Moreover, our analysis indicated that the low-risk group exhibited increased DC infiltration, heightened expression of antigen presentation-related signals, and elevated infiltration of various T cell types, including CD8 + T cells, tumor-infiltrating lymphocytes, and regulatory T cells (Treg). Intriguingly, VD metabolic pathways typically induce antigen-presenting cell immaturity and tolerance by inhibiting the expression of major histocompatibility complex II and costimulatory molecules on their surface, leading to reduced antigen-presenting inflammatory responses [35]. Furthermore, 1,25(OH)_2_D, a product of the VD metabolic pathway produced by mononuclear phagocytes, can inhibit T lymphocyte proliferation, promote the phenotypic transformation of Th1 and Th17 cells into Th2 cells, and induce Treg differentiation, thereby suppressing the pro-inflammatory state [36]. These disparities suggest that the effects of VD metabolic pathways on the PCa TME may deviate from the normal physiological state and require further investigation.

The mutation landscape of a tumor is closely intertwined with metabolic reprogramming. Our analysis indicated significantly higher mutation rates of TP53 and FOXA1 in the high-risk group. TP53 is a classical tumor suppressor gene implicated in numerous cancers; however, its association with the VD metabolism pathway has received limited attention. In the context of lung cancer, VD derivatives exhibit stronger antiproliferative activity against lung cancers harboring TP53 deletion mutations [37]. Whether TP53 mutations influence the antitumor effects of VD derivatives in PCa remains an unexplored area of research. FOXA1, encoding a transcription factor that regulates PCa biological behavior through AR-dependent and AR-independent pathways, is one of the most commonly mutated genes in PCa [38]. Our analysis suggests that the induction of dysregulated VD metabolism may represent a potential mechanism by which FOXA1 regulates PCa. Previous studies have shown that TP53 missense mutations are significantly enriched in PCa, which is the most common type of mutation included in the cohort in our study. It often occurs in the DNA-binding domain of the p53 gene, and the most common mutation is at the R273 residue, which can cause the loss of tumor suppressor function of wild-type p53 [39]. The missense mutation of TP53 is also associated with an increase in tumor infiltrating T cells within the TME of PCa [40]. The high incidence of nonsense mutations in the high-risk group found in this study can also lead to the loss of TP53 function, which is significantly associated with the stronger invasiveness of PCa [41]. However, whether these mutations alter the function of the TP53 protein in PCa by affecting its structure has not been revealed.

In our exploration of the potential therapeutic strategies related to VD metabolic activity, we observed intriguing findings. High-risk patients appeared to exhibit resistance to EGFR inhibitors and increased sensitivity to MAPK inhibitors. Interestingly, the enrichment analysis of intersection genes between vitamin D metabolism-related gene modules and differential genes in PCa and para-cancer tissues also suggested that these genes may be associated with MAPK signaling pathways (Fig. 2F). Moreover, our analysis suggested that the high-risk group may have reduced sensitivity to PARP inhibitors, which applied to metastatic CRPC patients carrying BRCA1/2 mutations [42].

Subsequently, we selected APOE as a potential therapeutic target and confirmed its differential expression and significant pro-cancer function using in vitro experiments. APOE is a multifunctional protein involved in lipid transport and lipoprotein metabolism with genetic polymorphisms (ε2, ε3, and ε4). It has been linked to various degenerative diseases such as coronary heart disease and Alzheimer’s disease. In the context of neurodegenerative diseases, VD deficiency is significantly associated with disease occurrence, and low serum levels of VD in conjunction with the APOE ε4 allele can synergistically affect disease progression [43, 44]. A cross-sectional study also established a significant association between the APOE ε4 allele and elevated serum VD levels [45]. Recently, APOE has garnered attention in the field of cancer. It is highly expressed in various solid tumors, including PCa, and is implicated in numerous tumor-related processes such as proliferation, invasion, and remodeling of the TME [46]. Previous studies have demonstrated that the ε2 and ε4 alleles of APOE can induce cholesterol overload in PCa cells, resulting in upregulation of cav1 expression, which in turn promotes PCa invasiveness [47]. Furthermore, recent research has revealed that APOE secreted by PCa cells can bind to TREM2 on the surface of neutrophils, inducing senescence and contributing to the establishment of an immunosuppressive TME [48]. However, the impact of APOE on the intrinsic biological characteristics of PCa requires further exploration, and its potential connection with the VD metabolic pathway remains unknown.

## Conclusions

In this study, we investigated genes associated with VD metabolic activity in PCa and constructed a robust prognostic model with strong predictive capabilities based on these genes. Utilizing this model, we investigated the relationship between dysregulated VD metabolism and aspects of PCa, including TME, mutational landscape, and sensitivity to multiple drugs. Finally, we validated the potential therapeutic targets identified through our analysis via in vitro experiments. Nevertheless, it is essential to acknowledge the limitations of our study. Nearly all of our findings were derived from data analysis, and their validity requires further confirmation through in vivo and in vitro experiments. The interaction between APOE and the VD metabolic pathway, as well as the mechanisms underlying the regulation of PCa’s biological characteristics of PCa, remain partially unexplained and represent important areas for future research. In conclusion, our study contributes to our understanding of how the VD metabolic pathway regulates PCa and provides a foundation for further comprehensive investigations, potentially leading to novel combination therapeutic strategies for metastatic PCa.

### Electronic supplementary material

Below is the link to the electronic supplementary material.

Supplementary Material 1

## Data Availability

All data comes from publicly available databases. We annotated the data sources in the article. For further information, please email the corresponding author.

## Abbreviations

aDCs: Activated dendritic cells
APC: Antigen presenting cell
APOE: Apolipoprotein E
AR: Androgen receptor
CCR: CC chemokine receptor
DCs: Dendritic cells
DEGs: Differentially expressed genes
Del: Deletion
DFS: Disease-free survival
FPKM: Fragments Per Kilobase of exon model
GEO: Gene Expression Omnibus
GO: Gene Ontology
GSEA: Gene Set Enrichment Analysis
HLA: Human leukocyte antigen
IC50: Half-maximal inhibitory concentration
iDCs: Immature dendritic cells
IFN: Interferon
Ins: Insertion
K-M: Kaplan-Meier
KEGG: Kyoto Encyclopedia of Genes and Genomes
MHC: Major histocompatibility complex
MSI: Microsatellite instability
PCa: Prostate cancer
pDCs: Plasmacytoid dendritic cells
PSA: Prostate-specific antigen
ROC: Receiver operating characteristic
SNP: Single nucleotide polymorphisms
TCGA: The Cancer Genome Atlas
Tfh: Follicular helper T cells
TIL: Tumor-infiltrating lymphocyte
TMB: Tumor mutation burden
TME: Tumor microenvironment
TPM: Transcripts Per Kilobase of exon model
Treg: Regulatory T cell
VD: Vitamin D
VDR: Vitamin D receptor
WGCNA: Weighted gene co-expression network analysis
