# Supplementary material for: Exploration of vitamin D metabolic activity-related biological effects and corresponding therapeutic targets in prostate cancer
Source: Nutr Metab (Lond). 2024 Apr 2;21:17. doi: 10.1186/s12986-024-00791-2 (PMC10988890; doi:10.1186/s12986-024-00791-2)
Supplement: Supplementary file 1 — Supplementary Material 1 [file 12986_2024_791_MOESM1_ESM.docx]

**Supplementary methods and materials**

**Supplementary methods**

**Cell culture and cell transfection**

Human PCa cell line C4-2 was purchased from the Chinese Academy of Sciences (Shanghai, China) and cultured in RMI1640 (Gibco, USA) medium containing 10% foetal bovine serum. They were placed in a humidifying incubator containing 5%CO2 at 37℃. The PCa cells were transfected with non-specific miRNA control and APOE siRNA (Hanbio, Shanghai, China) by using Lipofectamine 3000 (Invitrogen, USA) according to the manufacturer’s protocol. The transfection sequence used in the experiment was: si-APOE-2 (5’- GAAGGAGUUGAAGGCCUACAATT -3’).

**Quantitative real-time polymerase chain reaction (qRT-PCR)**

Using Trizol (Invitrogen, USA) for RNA extraction from the PCa cells according to the manufacturer’s instruction. MiRNA was reverse-transcribed into cDNA using MiR-XTM miRNA First-Strand Synthesis (Takara, JPN). The total RNA was reversed transcribed into cDNA using PrimeScript RT Master Mix (Takara, JPN). A standard SYBR Green PCR kit (Takara, JPN) was used to perform qRT-PCR. The following forward and reverse primer sequences of APOE are 5’- TGGAGCAAGCGGTGGAGACAG-3’ and 5’- CTCCTCCTGCACCTGCTCAGAC-3’, respectively.

**Western blotting**

The total proteins of the PCa cells were lysed in RIPA buffer (KeyGene biotech) supplemented with protease inhibitors. Protein was separated by 10% SDS/PAGE after boiling the samples for 15 min. Then the lysates were transfected onto PVDF membranes in transfer buffer. The PVD F membranes were blocked in 5% non-fat milk with Tris-buffered saline with Tween (TBST) for 3 h, then the PVDF membranes were treated overnight at 4°C with the following primary antibodies: APOE (1:5000), GAPDH (1:10000) (Abcam, UK). After cleaning with TBST, the PVD F membranes were treated with the secondary antibody (Abcam, UK) of the corresponding species, and finally exposed on the ECL luminometer and collected the image.

**Cell counting kit-8 (CCK-8) assay and cytotoxicity experiment**

For CCK-8 assay, we placed cancer cells into the 96-well plate (2.0*10^3^ per well), and 10 μl CCK-8 solution (Beyotime, China) was added into each well at 4, 24, 48, 72 and 96 h. The cells were then incubated at 37°C for 2h and then measured the optical density at 450 nm by an absorbance reader (Thermo Scientific, USA). And for cytotoxicity experiments, we also placed cancer cells into the 96-well plate (2.0*10^3^ per well). When the cells were attached to the wall, enzalutamide was added according to the established concentration gradient. 10 μl CCK-8 solution was added into each well at 72 h. The optical density at 450nm was read to calculate the inhibitory rate of the cells.

**Cell migration assay and invasion assay**

The Transwell upper chambers coated with or without matrigel were added with 200 ul serum-free medium and 3*10^5^ cancer cells, and then they were placed in a 24-well plate containing 800 ul medium containing 20% fetal bovine serum in each well and cultured at 37℃ for 48h. The Transwell upper chambers were then rinsed with PBS to remove unmigrated cells and fixed with 4% paraformaldehyde before staining with haematoxylin. Finally, they were observed and photographed under the optical microscope.

**5-Ethynyl-2’-deoxyuridine cell proliferation assay**

The Cell-Light KFluor555 EdU kit (KeyGEN, China) was used to monitor cell proliferation status. For the EdU assay, transfected cells (4 × 104) were plated in 24-well plates. When the cells cover 80% of the bottom of the well, C4-2 cells were treated for 3h with EdU solution (10 µM). After fixation with 4% formaldehyde (Servicebio), 0.5% Triton X-100 was applied to infiltrate the cells. The Click-iT reaction mixture was used to stain the cells, and counterstaining was performed using DAPI (Beyotime, China). Five random fields were photographed via the fluorescence microscope.

**Supplementary figures**


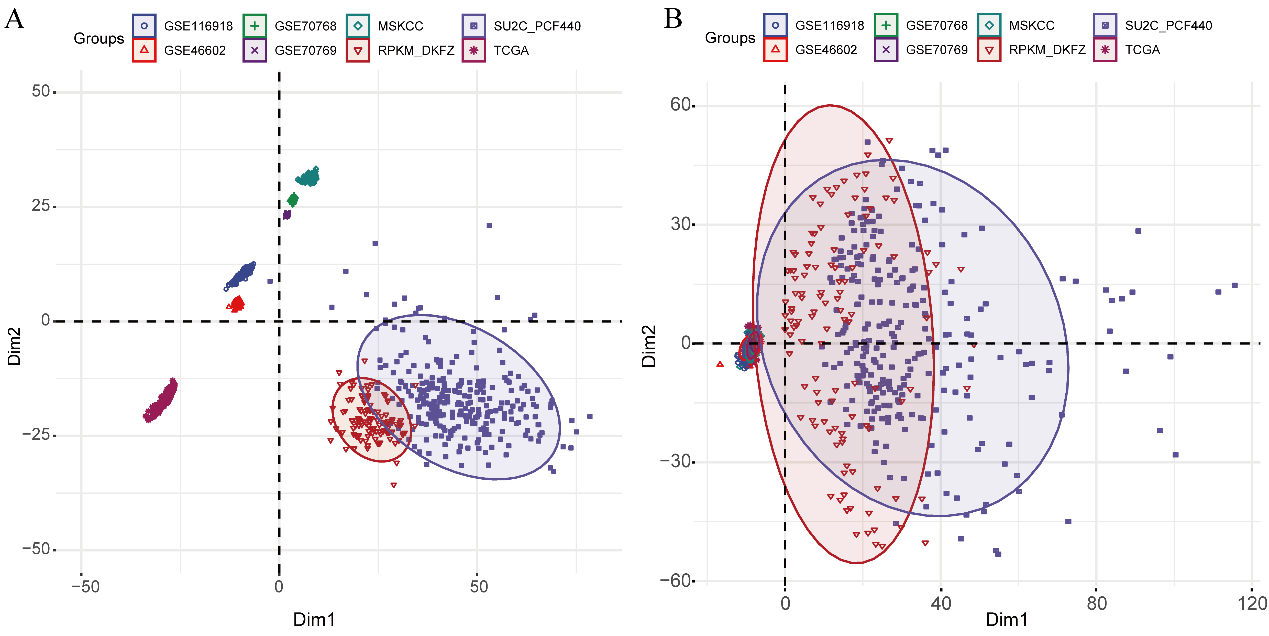


**Figure.S1 Demonstration of batch effect removal.** The expression of genes in all datasets was visualized based on principal component analysis before (A) and after (B) batch effect removal.


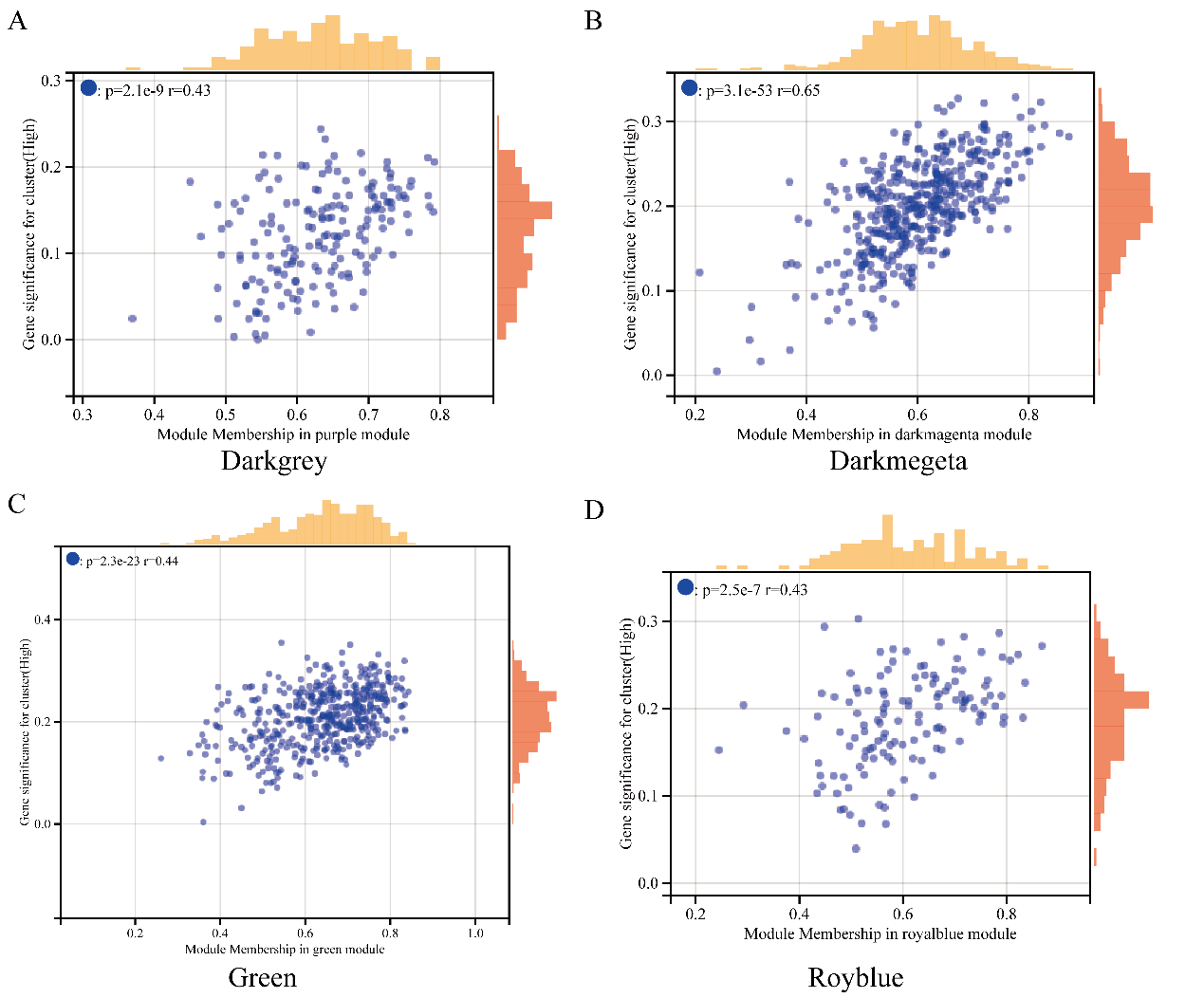


**Figure.S2 Demonstration of correlation between gene modules and vitamin D metabolic activity.** Point plot of correlation analysis of module membership (correlation between module genes and modules) and gene significance (correlation between module genes and vitamin D active phenotype) in the module of Darkgrey (A), Darkmegeta(B), Green(C), Royblue(D), reflecting whether the genes highly associated with the trait were also important genes in the corresponding module of the trait.


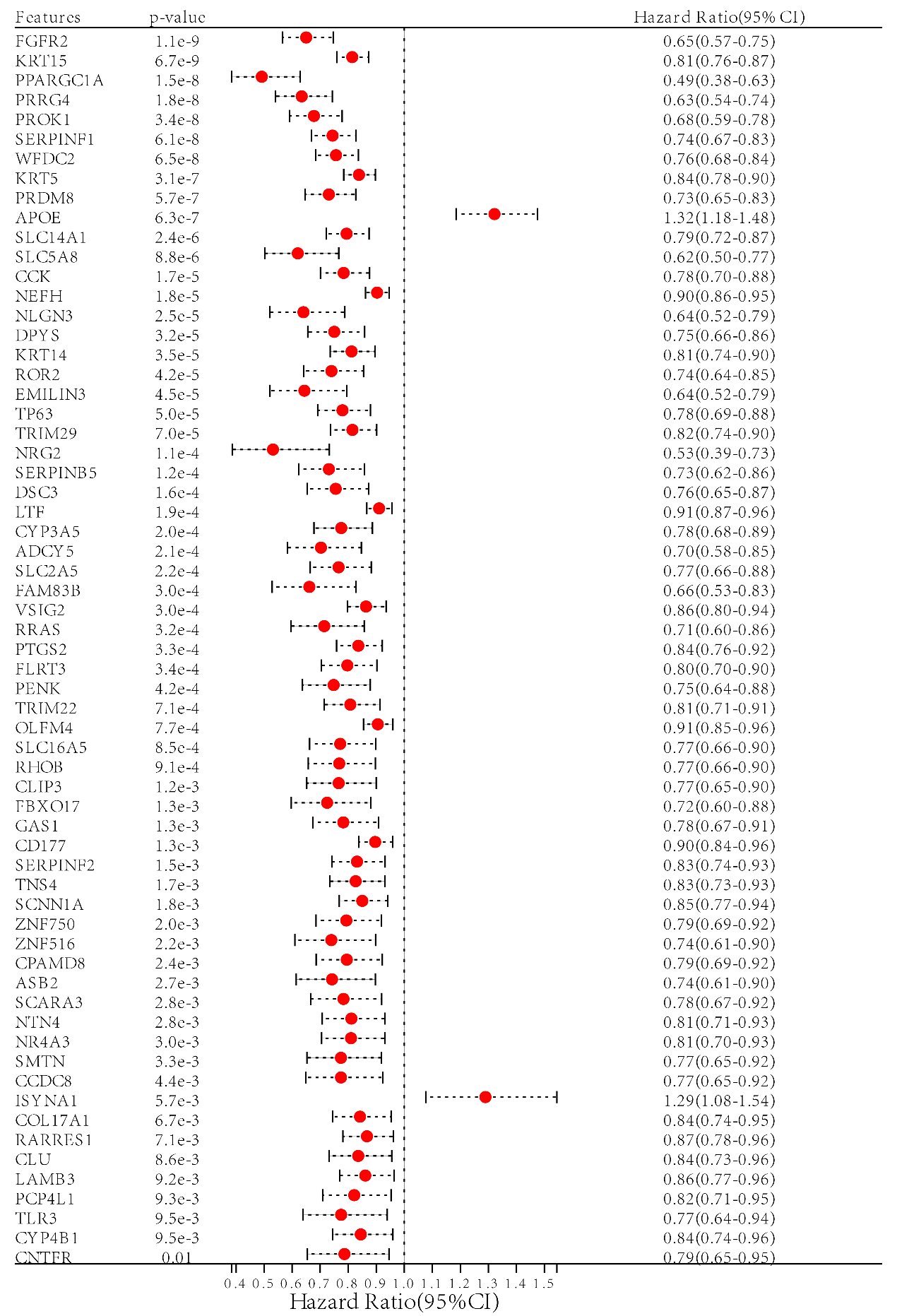


**Figure.S3 Screening of prognostic related genes.** The forest map exhibits the results of univariate regression analyses of prognostic related genes in intersection genes in Figure.2C.
